# Significantly increased anti‐tumor activity of carcinoembryonic antigen‐specific chimeric antigen receptor T cells in combination with recombinant human IL‐12

**DOI:** 10.1002/cam4.2361

**Published:** 2019-06-25

**Authors:** Xiaowei Chi, Peiwei Yang, Erhao Zhang, Jieyi Gu, Hui Xu, Mengwei Li, Xinmei Gao, Xin Li, Yinan Zhang, Hanmei Xu, Jialiang Hu

**Affiliations:** ^1^ The Engineering Research Center of Synthetic Polypeptide Drug Discovery and Evaluation of Jiangsu Province Nanjing P. R. China; ^2^ State Key Laboratory of Natural Medicines, Ministry of Education China Pharmaceutical University Nanjing P. R. China; ^3^ Development Center for Medicine Science and Technology National Health and Family Planning Commission of the People's Republic of China Beijing P. R. China

**Keywords:** carcinoembryonic antigen, chimeric antigen receptor T cell, colorectal cancer, gastric cancer, IL‐12, pancreatic cancer, solid tumor

## Abstract

**Background aims:**

Chimeric antigen receptor T cells (CAR‐T cells) have been successfully used in treatments of hematological tumors, however, their anti‐tumor activity in solid tumor treatments was limited. As IL‐12 increases T‐cell immune functions, we designed carcinoembryonic antigen (CEA) specific CAR‐T (CEA‐CAR‐T) cells and, for the first time, used them in combination with recombinant human IL‐12 (rhIL‐12) to treat several types of solid tumors.

**Methods:**

In vitro anti‐tumor activity of CEA‐CAR‐T cells in combination with rhIL‐12 was confirmed by evaluation of CEA‐CAR‐T cell activation, proliferation, and cytotoxicity after co‐incubation with CEA‐positive or CEA‐negative human tumor cells. In vivo anti‐tumor activity of CEA‐CAR‐T cells in combination with rhIL‐12 was confirmed in a xenograft model in nude mice for treatments of several types of solid tumors.

**Results:**

In vitro experiments confirmed that rhIL‐12 significantly increased the activation, proliferation, and cytotoxicity of CEA‐CAR‐T cells. Similarly, in vivo experiments found that CEA‐CAR‐T cells in combination with rhIL‐12 had significantly enhanced anti‐tumor activity than CEA‐CAR‐T cells in growth inhibition of newly colonized colorectal cancer cell HT‐29, pancreatic cancer cell AsPC‐1, and gastric cancer cell MGC803.

**Conclusions:**

These works confirmed that simultaneous use of cytokines, for example, rhIL‐12, can increase the anti‐tumor activity of CAR‐T cells, especially for treatments of several types of solid tumors.

## 1 INTRODUCTION

In recent years, chimeric antigen receptor T (CAR‐T) cells have been used as a new way of cancer treatment.^1^ Using CAR‐T cells to treat B‐cell malignances, especially acute lymphocyte leukemia, has achieved promising clinical results.^2^ Although promising results were achieved in CAR‐T cell treatments of hematological cancer, the efficacy of their treatments of solid tumors is limited.^3^ Many reasons exist for the limited efficacy, of which the components of tumor microenvironments (TME) remains the main obstacle.^4^ The established solid tumor tissue includes not only tumor cells but also inhibitory immune cells (eg, regulatory T cells, tumor‐associated macrophages, myeloid‐derived suppressor cells), inhibitory cytokines (eg, IL‐4, IL‐6, IL‐10, and TGF‐β), inhibitory immune molecules (eg, PD‐1, CTLA‐4, LAG3, and Tim3) and other inhibitory factors (eg, oxidative stress, lack of nutrients, acidic pH and hypoxia) and all these factors form TME.^5^, ^6^ TME decreases immune cell infiltration and inhibits their functions which leads to tumor immune escape.^7^ New treatment strategies are urgently needed to aid CAR‐T cells to treat solid tumors within this TME. Normally, the concentrations of cytokines that are the main components for T‐cell activation, proliferation, and cytotoxicity are low in TME.^8^ Preclinical studies have confirmed that the usage of cytokines such as IL‐2, IL‐7, IL‐12, IL‐15, IL‐21 showed anti‐tumor activity.^9^, ^10^, ^11^ Of these immune stimulating cytokines, IL‐12 is a central cytokine and the most promising candidate in cancer treatment.^12^ Research has found that IL‐12 can induce the formation of Th1 and Th17 cells,^13^ increase the secretion of IFN‐γ and the cytotoxic effect of NK and T cells,^14^ recruit and activate innate immune cells, strengthen antigen cross presentation, and re‐edit T regulatory cells.^15^ These functions contributed to the confirmed anti‐tumor activity of ectogenic IL‐12 in treatment of solid tumors.^16^, ^17^ Recently, Oladapo O. Yeku et al designed a CAR‐T cell that can constitutively express IL‐12. Secreted IL‐12 helped the CAR‐T cells to overcome the hostile TME and strengthen their anti‐tumor activity.^18^ However, other research found that constitutive expression of IL‐12 led to nonspecific activation of CAR‐T cells which also caused serious side effect.^19^

In this study, we propose that combination use of rhIL‐12 and CAR‐T cells can enhance the anti‐tumor activity of CAR‐T cells. As a tumor‐associated antigen, carcinoembryonic antigen (CEA) is expressed in a polarized way in health cells whereas it is expressed on the whole cell surface of cancer cells. CEA has been found to be expressed on several types of cancer, for example, colorectal cancer, pancreatic cancer, gastric cancer, lung cancer, breast cancer, and its expression is correlated with the degree of cancer malignancy.^20^ Therefore, we designed CEA‐specific CAR‐T cells and evaluated their efficacy in solid tumor treatment when in combination use with rhIL‐12. The results confirmed that combination use of CEA‐CAR‐T cells and rhIL‐12 showed significantly enhanced anti‐tumor activity against in vivo growth of colorectal cancer cell HT‐29, pancreatic cancer cell AsPC‐1 and gastric cancer cell MGC803 than single use of CEA‐CAR‐T cells. These results show that combination use of CEA‐CAR‐T cells and rhIL‐12 can overcome the limitation of their single use as anti‐cancer drugs and provide a new strategy for solid cancer treatment.

## 2 MATERIALS AND METHODS

### 2.1 Cell lines and culture conditions

Fresh blood was collected from healthy volunteers after obtaining informed consent from the review committee of China Pharmaceutical University. Peripheral blood mononuclear cells were isolated from fresh blood by gradient centrifugation with use of Lymphoprep^™^ (Axis‐Shield, Norseland). T cells were selectively enriched with CD3^+^ separation beads (Miltenyi Biotec Inc, Auburn, CA, USA). Isolated T cells were cultured in X‐VIVO15 culture medium (Lonza, Switzerland) supplemented with 5% human AB serum (Valley Biomedical Inc, Winchester, VA, USA), 10 mM N‐acetyl l‐cysteine (Sigma Aldrich, St. Louis, MO, USA), and 300 IU/mL recombinant human IL‐2 (PeproTech, Rocky Hill, CT, USA).

Human pancreatic cancer cell AsPC‐1 and BxPC‐3, colorectal cancer cell HT‐29 and gastric cancer cell MGC803 were obtained from American Type Culture Collection. AsPC‐1 cells were cultured in RPMI1640 culture (Hyclone, Logan, UT, USA) and PANC‐1, HT‐29, and MGC‐803 cells were cultured in DMEM culture (Hyclone). All the tumor cultures were supplemented with 10% fetal bovine serum (FBS) (Gibco, Gaithersburg, MD, USA), 2 mmol/L Glutamine (Gibco), 100 U/mL penicillin, and 100 µg/mL streptomycin (Sangong Biotech, Shanghai, China).

### 2.2 Construction of plasmid

Based on pLV‐puro carrier (Hanbio Biotechnology Co., LTD, Shanghai, China), various lentiviral carriers encoding CAR and red fluorescence protein (RFP) were constructed. In brief, for CEA‐CAR‐T cell construction, CEA‐CAR plasmid contains the following gene elements (from 5′ end to 3′ end): Xho Isite, Kozak and CD8 signal peptide, anti‐CEA scFv, hinge region and CD8α transmembrane domain, 4/1BB cytoplasm structure domain and CD3ζ, P2A and green fluorescence protein (GFP) sequence, and aXba I site. For target cell construction, RFP carrier contains the following elements (from 5′ end to 3′ end): Xho Isite, Kozak and signal peptide sequence, RFP sequence and Xba Isite. The CEA scFV in the CAR carrier was kindly provided by Prof. Hanmei Xu in China Pharmaceutical University. The sequence of the other gene elements was obtained from National Biotechnology Information Center. After codon optimization, the DNA molecules of the carriers were synthesized by GENEWIZ (Suzhou, China) and the synthesized DNA molecules were cut and incoporated into pLV‐puro carrier by Xho I and Xba I sites.

### 2.3 Construction of T cells and target cells by lentiviral transfection

Freshly isolated human T cells were incubated with CD3/CD28 magnetic beads (Invitrogen, Carlsbad, CA, USA) at a ratio of 3:1 beads:cells. After T‐cell activation for 48 hours, engineered lentivirus was added into the cell culture with a MOI of 15 and polybrene was also added with a final concentration of 6 µg/mL. The cells were incubated at 37°C in 5% CO_2_ overnight. After viral infection for 5 days, T cells were collected and their expression of CAR were analyzed and confirmed by flow cytometry analysis and western blot analysis.

For target cell transfection, AsPC‐1, BxPC‐3, HT‐29, and MGC803 cells were cultured to logarithmic growth phase. The cells were collected and added to a six well plate and incubated in presence of 6 µg/mL polybrene and proper amount of lentivirus in fresh culture medium. After incubation for 24 hours, culture supernatant was replaced with fresh culture medium. After incubation for 5 days, puromycin was used to select RFP expressing tumor cells. The expression of RFP was confirmed by flow cytometry analysis and western blot analysis.

### 2.4 Flow cytometry analysis and western blot analysis

For flow cytometry analysis, all the cells were collected by centrifugation and were washed by FACS washing buffer that contains 0.5% BSA and 0.03% sodium azide for three times. Anti‐CEA monoclonal antibody (BD, San Jose, CA, USA) was used to detect CEA using the FITC channel. For T‐cell activation detection, after overnight activation, the activation marker CD25 and CD69 were detected with APC‐conjugated anti‐CD25 or anti‐CD69 antibody (Biolegend, San Diego,CA). Anti‐CD4 (BD, APC‐conjugated) and anti‐CD8 (BD, PE‐conjugated) antibodies were used to examine the phenotype of T cells in vivo. Antibody was incubated with the cells for 30 minutes at 4°C in dark. Finally, cells were washed and detected.

Traditional western blot was performed to detect GFP and RFP expression. Protein samples were extracted from lyzed cells and kept in −80°C refrigerator until use. The first antibodies for GFP or RFP detection were from abcam (Cambridge, UK).

### 2.5 Enzyme‐linked immunosorbent assays

For enzyme‐linked immunosorbent assays (ELISA) in in vitro experiments, CEA‐CAR‐T cells and target cells were co‐cultured at a ratio of 2:1 in a 96‐well round bottom plate. After overnight incubation, supernatant was collected and IL‐2 and IFN‐γ were measured with an ELISA kit (MultiSciences, Hangzhou, China). For ELISA in in vivo experiments, 100 µL of blood was collected from experimental mice at indicated time points and levels of cytokines were measured with an ELISA kit (MultiSciences).

### 2.6 Quantitation of T‐cell proliferation

Tumor cells were treated with 10 µg/mL Mitomycin C (Sigma Aldrich) for 2 hours. Then 5 × 10^4^ Mitomycin C‐treated tumor cells were co‐cultured with four types of effector cells individually with each sample containing 1 × 10^5^ effector cells. The density of T cells was 5 × 10^5^/mL. After 7 days’ of co‐culture, cell counting for all the samples was performed and the positive ratio of CAR‐T cells in the CEA‐CAR‐T treatment samples was determined. IL‐12 was the only exogenous cytokines added during the proliferation assays.

### 2.7 Cytotoxicity assays

Carcinoembryonic antigen‐specific CAR‐T cells were co‐cultured with target cells at a ratio of 2:1 in 10% FBS‐containing T‐cell culture at 37°C for 24 hours. The cytotoxicity of CEA‐CAR‐T cells to target cells was evaluated by measuring lactic dehydrogenase (LDH) levels in cell culture supernatant with a LDH kit (Cayman, Ann Arbor, MI, USA). Experimental samples and control samples were set according to the manufacturer's suggestion. LDH levels in the samples were spectrometrically detected at a wavelength of 490 nm with a Multiscan FC plate reader (Thermo Scientific, Waltham, MA, USA). Finally, T‐cell cytotoxicity was calculated by the following formula: specific cytotoxicity (%) = (mixture cell experiment‐effector cell spontaneous‐target cell spontaneous‐medium control)/(target cell maximum‐target cell spontaneous‐medium control) × 100%.

### 2.8 Xenograft mouse models and in vivo imaging

Female Balb/c nude mice of 7‐9 weeks were kept in the Animal Center of China Pharmaceutical University. All animals were housed in a controlled environment (25°C; 12 hours light‐dark cycle), with water and food provided freely. The authors confirm that experiments involving animals adhered to the institutional ethical standards of China Pharmaceutical University and the care of animals was independently assessed and approved in accordance with the licensing guidelines of China Pharmaceutical University.

At the beginning, mice were put in four groups with each group innoculated with one type of the tumor cells. For the mice injected with CEA‐positive AsPC‐1‐RFP, HT‐29‐RFP, or MGC803‐RFP cells, they are further put in four groups. For the mice injected with CEA‐negative BxPC‐3‐RFP cells, they are further put in two groups. All the mice were intravenously injected with 5 × 10^6^ engineered tumor cells on day 0. Seven days after tumor cell injections, we measured fluorescent signal for each mouse and based on this data mice are grouped so that the difference in total fluorescent signal strength between different groups is minimal. At day 7 after tumor cell innoculation, each mouse was intravenously injected with 1 × 10^7^ CEA‐CAR‐T cells or untransduced T cells. For the mice in the group with rhIL‐12 (PeproTech) treatment, each mouse was intravenously injected with 1500 U/mouse rhIL‐12 at day 7, 9 ,12, 15, 19, and 25 according to the literature.^21^, ^22^

To detect the anti‐tumor activity of CEA‐CAR‐T cells, tumor burden of each mice was measured by detection of the fluorescence signal with an in vivo imaging system (Fx Pro, Carestream Health,USA) at day 7, 14, 21, 28, and 35. T‐cell cytotoxicity was calculated according to fluorescence signal strength emitted from RFP expressing tumor cells in tumor tissue. Finally, relative cytotoxicity of engineered T cells was calculated at day 35 by comparison of average fluorescence signal strength in engineered T cell inoculated mice with average fluorescence signal strength in untransduced T cell inoculated mice.

### 2.9 Quantitation of T‐cell counts and cytokine production in vivo

In the same animal experiment as described above, 100 µL of blood was collected from mice at day 21 after inoculation of tumor cells to measure in vivo T‐cell proliferation. T‐cell counts were quantified for the collected blood samples. At the end of the animal experiment, levels of cytokines such as IL‐2, IL‐12, IFN‐γ, and TNF‐α in blood samples from each mouse were measured with ELISA assays.

### 2.10 Statistical analysis

Statistical significance was determined by Student's *t* test (two‐tailed). All statistical analysis was performed with GraphPad Prism 7. All error bars represent either SEM or SD.

## 3 RESULTS

### 3.1 Construction of antigen‐specific CAR‐T cells and fluorescence generating target cells

**Figure 1:**
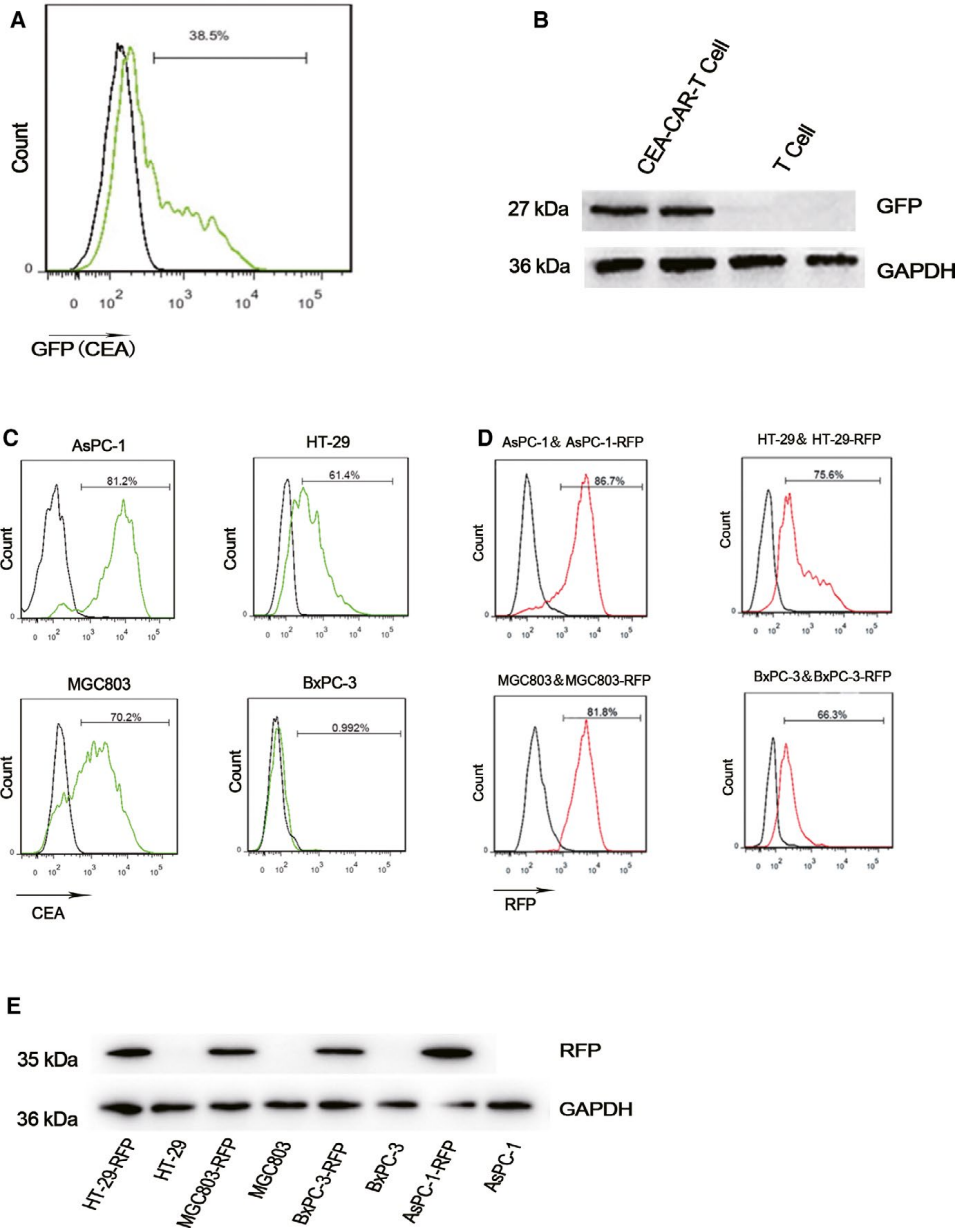
Construction of CEA‐specific CAR‐T cells and target tumor cells. (A) Flow cytometry detection of green fluorescence protein (GFP) expression by CEA‐CAR‐T cells to evaluate their transfection rate. (B) Western blot analysis of GFP expression in CEA‐CAR‐T cells. GAPDH as a loading control is at 36kD in all lanes. (C) Flow cytometry analysis of CEA levels on target tumor cells. (D) Flow cytometry analysis of reporter protein red fluorescence protein (RFP) levels to evaluate lentiviral transfection of tumor cells. (E) Western blot analysis of RFP expression in tumor cells. GAPDH as a loading control. CAR‐T, chimeric antigen receptor T

We constructed a second‐generation CEA targeting CAR, in which CD3ζ induces T‐cell activation and 4‐1BB behaves as a co‐stimulator. A GFP reporter protein was inserted in CAR sequence which helps to detect T cells which are successfully transduced and express CAR. After lentiviral infection, flow cytometry analysis (Figure 1A), and western blot analysis (Figure 1B) confirmed GFP expression and successful CAR transduction in T cells with untransduced T cells as a negative control cell. Ratios of CAR‐positive T cells for the four time CEA‐T cell construction were shown in Figure S1.

We selected colorectal cancer cell HT‐29, pancreatic cancer cell AsPC‐1, and gastric cancer cell MGC803 as target cells as they highly express CEA. A pancreatic cancer cell BxPC‐3 was used as a negative control cell as it does not express CEA (Figure 1C). For convenience of detection in the following experiments, these cells were genetically modified to express RFP by lentiviral infection. After antibiotics selection, transfected cells that stably express RFP were obtained with name HT‐29‐RFP, AsPC‐1‐RFP, MGC803‐RFP, and BxPC‐3‐RFP. Their expression of RFP was confirmed by flow cytometry analysis (Figure 1D) and western blot analysis (Figure 1E).

### 3.2 Optimal effector cell to target cell ratio of CEA‐CAR‐T cells and dose titration for rhIL‐12

**Figure 2:**
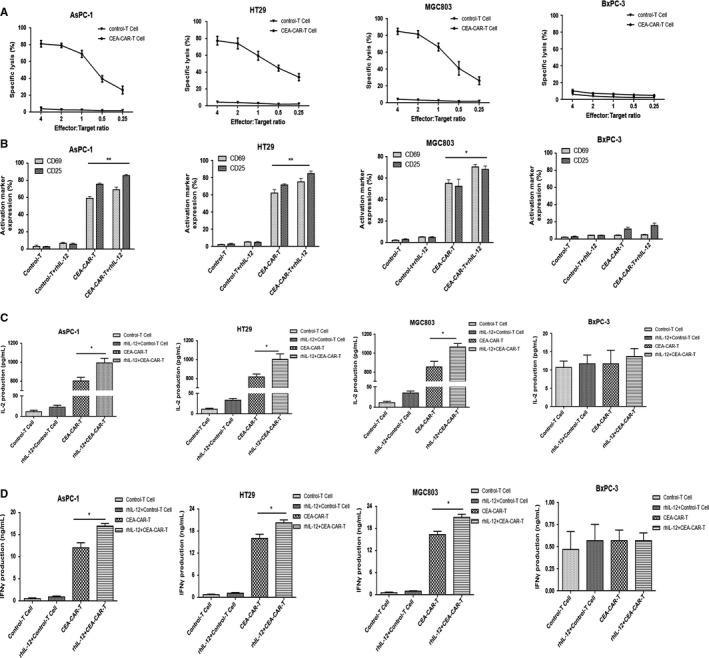
In vitro activation of CEA‐CAR‐T cells in combination with rhIL‐12. (A) 1 × 10^4^ tumor cells were put in each well of the plate. CEA‐CAR‐T cells or untransduced T cells were cocultured with target tumor cells at different effector to target ratios and the lactic dehydrogenase (LDH) levels in the culture supernatant were measured after 24 h. (B) 1 × 10^4^ tumor cells were put in each well of the plate at an effector cell to target cell ratio of 2:1 and the cells were cocultured for 24 h. Activation marker of CD25 and CD69 on T‐cell surface were detected with flow cytometry analysis (n = 3, error bars denote standard deviation,*P < 0.05, **P < 0.01). (C) 1 × 10^4^ tumor cells were put in each well of the plate at an effector cell to target cell ratio of 2:1 and the cells were co‐cultured for 24 h. Levels of IL‐2 secreted by T cells were measured (n = 3, error bars denote standard deviation, *P < 0.05). (D) 24 h after incubation of effector cells and target cells, levels of IFN‐γ secreted by T cells were measured (n = 3, error bars denote standard deviation, *P < 0.05). CAR‐T, chimeric antigen receptor T; CEA, carcinoembryonic antigen

In vitro cytotoxic experiment was performed to define a proper effector cell to target cell ratio for subsequent experiments. In this experiment, CEA‐CAR‐T cells and tumor cell HT‐29, AsPC‐1, or MGC803 were co‐cultured at an effector cell to target cell ratio of 4:1, 2:1, 1:1, 1:2, or 1:4. After an overnight incubation, supernatant of cell culture under each experimental condition was collected and LDH level was measured with an ELISA method to evaluate and compare the anti‐tumor effect of CEA‐CAR‐T cells under each effector cell to target cell ratio. The experimental results showed that with the increase of effector cell to target cell ratio, the cytotoxic effect of CEA‐CAR‐T cells to CEA‐positive HT‐29, AsPC‐1 or MGC803 cells increased correspondingly. And the LDH level or the cytotoxic effect of CEA‐CAR‐T cells at an effector cell to target cell ratio of 4:1 was similar to a ratio of 2:1 (Figure 2A). Therefore, an effector cell to target cell ratio of 2:1 was used for the following experiments.

A dose‐titration experiment for rhIL‐12 was performed with the CEA‐CAR‐T cell cytotoxicity experiment. In this experiment, effector cells and target cells were co‐cultured at a ratio of 2:1 and after 24 hours, LDH levels in the culture supernatant were measured. The target cells are CEA‐positive HT‐29 cells and the series of rhIL‐12 dose are 1, 10, 50, 100, 200, 500, and 1000 U/ml. Experiment results showed that within 50 U/mL, the cytotoxic effect of CAR‐T cells increased with the increase in rhIL‐12 dose. When rhIL‐12 dose is more than 50 U/mL, the cytotoxic effect of CAR‐T cells is close to saturation (Figure S2).

### 3.3 rhIL‐12 significantly enhanced activation of CEA‐CAR‐T cells in vitro

To evaluate the in vitro effect of rhIL‐12 on CEA‐CAR‐T cell activation, four experimental conditions were set in which cancer cells are incubated with control‐T cells, control‐T cells and rhIL‐12, CEA‐CAR‐T cells, and CEA‐CAR‐T cells and rhIL‐12, respectively. Cancer cells include CEA‐positive HT‐29, AsPC‐1 and MGC803 cells and CEA‐negative BxPC‐3 cells. Effector cells (CEA‐CAR‐T cells) and target cells (tumor cells) were cultured together at a ratio of 2:1 for 24 hours. In rhIL‐12 treatment groups, based on the results of dose titration experiment for rhIL‐12 and literatures, the dose of rhIL‐12 was set at 200 U/mL.^21^, ^22^ CEA‐CAR‐T cell activation was evaluated by their expression of cell surface markers CD25 and CD69 (Figure 2B), and their secretion of cytokine IL‐2 and IFN‐γ (Figure 2C,D).

Compared with CEA‐negative BxPC‐3 cells, in experiments with CEA‐positive HT‐29, AsPC‐1 and MGC803 cell as target cells, cytokine IL‐2 and IFN‐γ expression levels in CEA‐CAR‐T cell group and CEA‐CAR‐T cell in combination with rhIL‐12 group were much higher than control‐T cell group and control‐T cells in combination with rhIL‐12 group. Furthermore, cytokine expression levels of CEA‐CAR‐T cell in combination with rhIL‐12 group were significantly higher than CEA‐CAR‐T cell group (Figure 2C,D). Whereas in the experiment with CEA‐negative BxPC‐3 cells as target cells, CEA‐CAR‐T cells were not activated and there were no significant changes of cytokine levels among the four groups (Figure 2C,D). Experiments were also performed to detect cell surface expression of CD25 and CD69, the expression levels of which were upregulated after T‐cell activation. Similarly, in the experiments with CEA‐positive tumor cells as target cells, the expression levels of CD25 and CD69 on CEA‐CAR‐T cells surface under CEA‐CAR‐T cells in combination with rhIL‐12 treatment were significantly higher than CEA‐CAR‐T cell treatment. In experiment with CEA‐negative tumor cells as target cells, the expression levels of CD25 and CD69 had no significant changes among the four treatment conditions (Figure 2B). These results confirmed that rhIL‐12 can significantly enhance the activation of CEA‐CAR‐T cells in vitro.

### 3.4 rhIL‐12 promoted CEA‐CAR‐T cell proliferation in vitro

**Figure 3:**
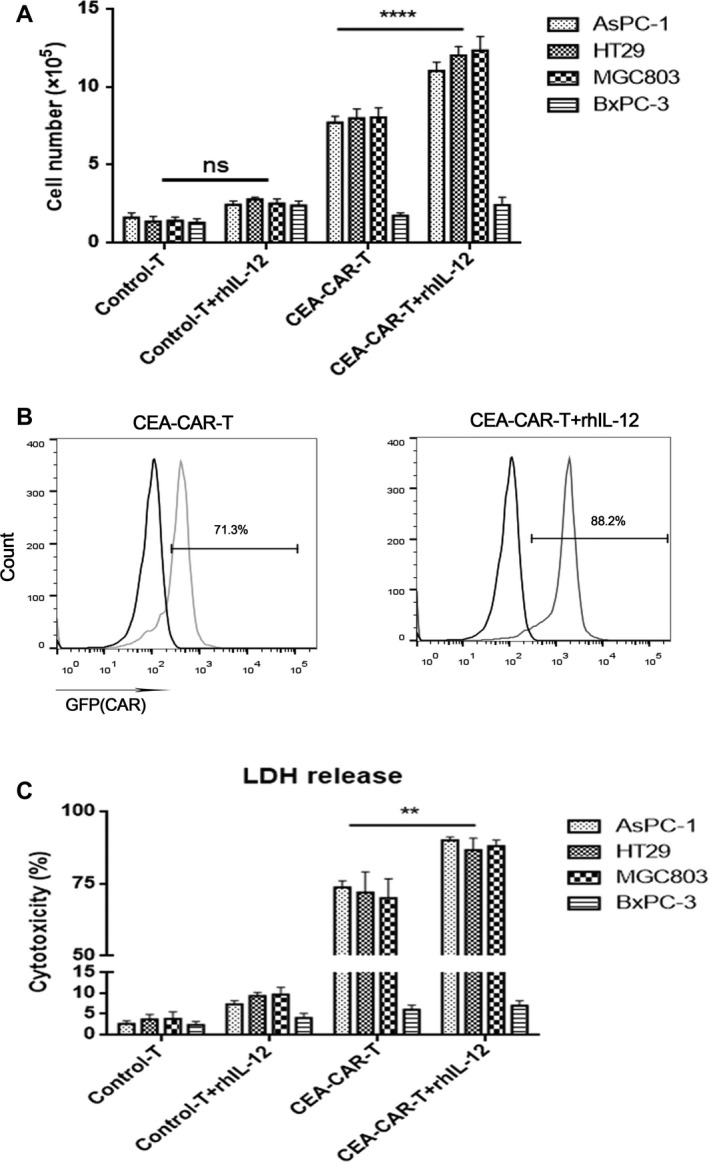
In vitro proliferation and cytotoxicity of CEA‐CAR‐T cells in combination with rhIL‐12. (A) Number of T cells in each sample in which 5 × 10^4^ Mitomycin C‐treated tumor cells were co‐cultured with 1 × 10^5^ effector cells for 7 d (n = 3, error bars denote standard deviation,****P < 0.0001). (B) The positive ratio of CAR‐T cells in CEA‐CAR‐T cell treatment groups after effector and target cell co‐culture for 7 d. (C) 1 × 10^4^ tumor cells were put in each well of the plate at an effector cell to target cell ratio of 2:1 and the cells were co‐cultured for 24 h. Levels of lactic dehydrogenase (LDH) in the supernatant of cell cocultures were measured (n = 3, error bars denote standard deviation, **P < 0.01). CAR‐T, chimeric antigen receptor T; CEA, carcinoembryonic antigen

Significantly increased CAR‐T cell proliferation after in vitro activation is an important parameter to evaluate CAR‐T cell activity. In this experiment, the four types of tumor cells after mitomycin C treatment were co‐cultured with control‐T cells, control‐T cells and rhIL‐12, CEA‐CAR‐T cells, and CEA‐CAR‐T cells and rhIL‐12. The dose of rhIL‐12 was 200 U/mL. After co‐culture for 7 days, T‐cell counting for all the samples was performed and the positive ratio of CAR‐T cells in the CEA‐CAR‐T treatment samples was determined. The experimental results showed that there was no significant change in T‐cell numbers when co‐cultured with CEA‐negative BxPC‐3 cells under the four treatment conditions whereas T‐cell number after co‐cultured with CEA‐positive tumor cells significantly increased under CEA‐CAR‐T treatment or CEA‐CAR‐T cells and rhIL‐12 treatment. T‐cell number under CEA‐CAR‐T cell and rhIL‐12 treatment was significantly more than CEA‐CAR‐T cell treatment when co‐culturing with each type of CEA‐positive tumor cells (Figure 3A). In addition, flow cytometry analysis showed that the positive ratio of CAR‐T cells was higher in rhIL‐12 treatment samples which confirmed that rhIL‐12 can induce CEA‐CAR‐T proliferation (Figure 3B). The experiment in Figure 3A,B confirmed that rhIL‐12 can promote the proliferation of CEA‐CAR‐T cells in presence of tumor cells that express the specific tumor‐associated antigen.

### 3.5 rhIL‐12 enhanced the cytotoxic effect of CEA‐CAR‐T cells

In the in vitro experiment, CAR‐T cells can specifically target and kill tumor cells and with this experimental system, we tested if rhIL‐12 can increase the cytotoxic effect of CAR‐T cells by measuring the supernatant LDH level that was released by tumor cells after their cytolysis. The experimental conditions and target tumor cells used were similar to the experiment shown in Figure 2C,D. Under control‐T cell or control‐T cells in combination with rhIL‐12 treatment, there was no significant changes in the levels of released LDH. Under CEA‐CAR‐T cell treatment or CEA‐CAR‐T cells in combination with rhIL‐12 treatment, there were significantly increased LDH levels in the supernatant of CEA‐positive tumor cell cultures and the LDH level under CEA‐CAR‐T cells in combination with rhIL‐12 treatment were significantly higher than CEA‐CAR‐T cells treatment. In addition, there were no significant changes in the LDH level in the supernatant of CEA‐negative BxPC‐3 cell culture and this LDH level was similar to the supernatant of BxPC‐3 under control‐T cell or control‐T cells in combination with rhIL‐12 treatment (Figure 3C). This in vitro experiment confirmed the specific cytotoxic effect of CEA‐CAR‐T cells and the enhanced cytotoxic effect after rhIL‐12 incubation.

### 3.6 In vivo anti‐tumor effect of CEA‐CAR‐T cells in combination with rhIL‐12

**Figure 4:**
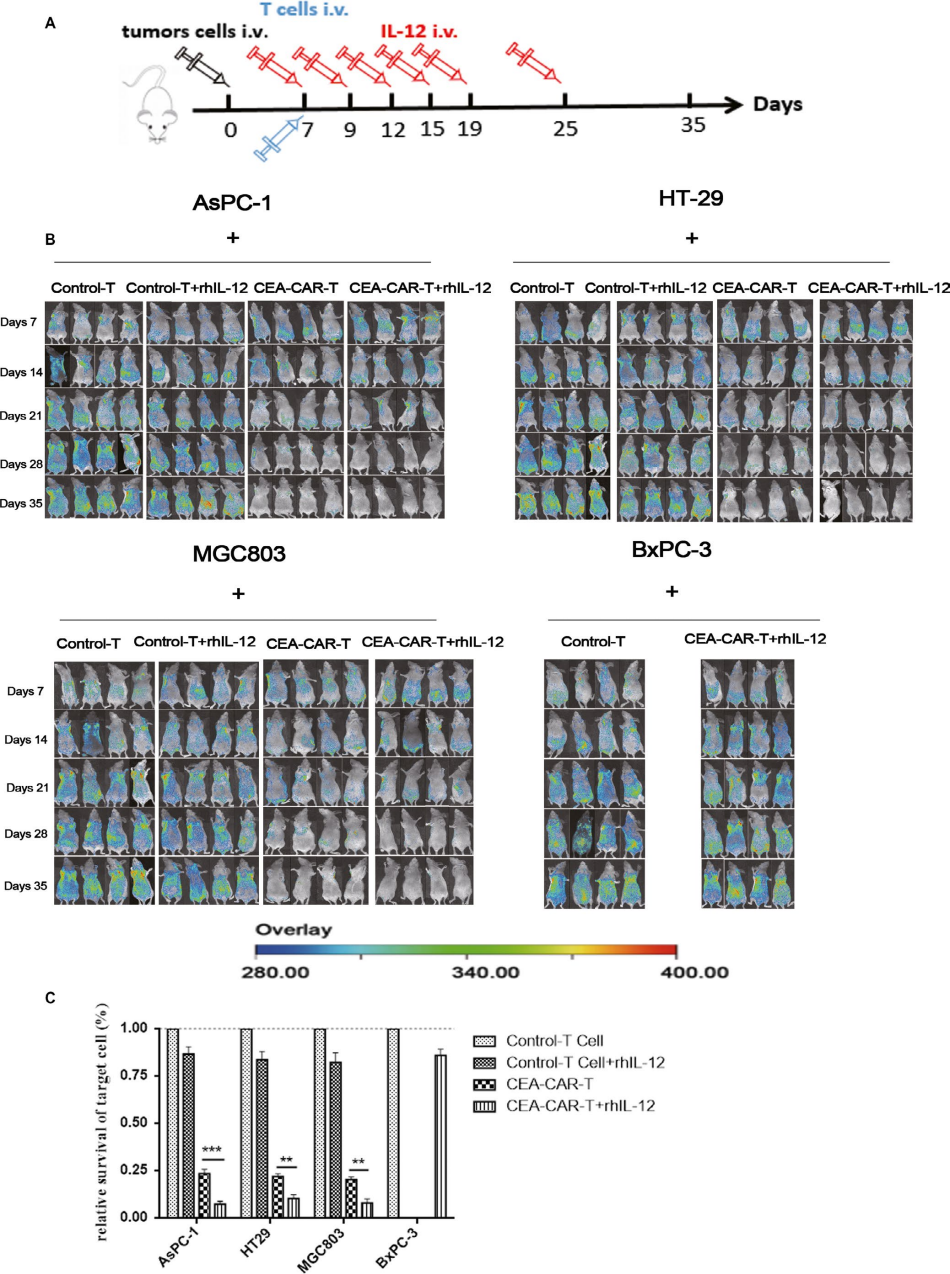
In vivo anti‐tumor activities of CEA‐CAR‐T cells in combination with rhIL‐12 on established xenografts. (A) Animal work strategy. (B) In vivo imaging of the fluorescence signal of mice at day 7, 14, 21, 28, and 35 after tumor cell inoculation (n = 4). (C) Survival rate of target cells in experimental mice 28 d after CEA‐CAR‐T cell inoculation (n = 4, error bars denote standard deviation, **, *P* < 0.01;***, *P* < 0.001). CAR‐T, chimeric antigen receptor T; CEA, carcinoembryonic antigen

To evaluate the in vivo anti‐tumor effect of CEA‐CAR‐T cells in combination with rhIL‐12, transfected tumor cell lines were used which express fluorescence reporter protein RFP. In this model, mice were intravenously injected with CEA‐positive HT‐29‐RFP, AsPC‐1‐RFP, MGC803‐RFP or CEA‐negative BxPC‐3‐RFP cells and each mouse received 5 × 10^6^ tumor cells. After 7 days, mice injected with CEA‐positive tumor cells were put into four groups. The mice in each group were treated with control‐T cells, control‐T cells and rhIL‐12, CEA‐CAR‐T cells or CEA‐CAR‐T cells and rhIL‐12. For mice injected with BxPC‐3‐RFP, we further divided the mice into two groups. The mice in each group were treated with control‐T cells or CEA‐CAR‐T cells in combination with rhIL‐12. 1 × 10^7^ CEA‐CAR‐T cells were intravenously injected into each mouse. RhIL‐12 was used according to the literatures^21^, ^22^ and 1500 U/mouse was intravenously injected at day 7, 9, 12, 15, 19, and 25 (Figure 4A). Fluorescence signal from RFP expressing tumor cells in mice were measured to follow the change of in vivo tumor growth after CEA‐CAR‐T cells treatment. Differences in fluorescent signal over time were provided in Figure S3. Based on the in vivo imaging result, in the mice that were intravenously injected with CEA‐positive HT‐29‐RFP, AsPC‐1‐RFP, or MGC803‐RFP cells and thereafter CEA‐CAR‐T cells 7 days later, the in vivo tumor generating fluorescence signal significantly decreased at 21 day after tumor cell inoculation, and the fluorescence signal decreased further at day 28. In the mice that were intravenously injected with CEA‐positive HT‐29‐RFP, AsPC‐1‐RFP or MGC803‐RFP cells and thereafter injected with CEA‐CAR‐T cells in combination with rhIL‐12 at day 7, the in vivo tumor generating fluorescence signal significantly decreased at 21 and almost disappeared at day 28. In the mice that were injected with CEA‐negative BxPC‐3 cells, CEA‐CAR‐T cells treatment or CEA‐CAR‐T cells in combination with rhIL‐12 treatment did not cause a decrease in in vivo fluorescence signal (Figure 4B).

Next, we used the average fluorescence signal of the mice in control‐T cell treatment group at day 35 to calibrate the relative cytotoxicity of the other treatment conditions (Figure 4C). The results showed that CEA‐CAR‐T cell treatment had significant anti‐tumor effect compared with control‐T cells. CEA‐CAR‐T cells in combination with rhIL‐12 had even stronger anti‐tumor effect than CEA‐CAR‐T cell treatment. Furthermore, no obvious body weight loss was found for mice in all treatment groups (Figure S4). The above in vitro experiment results were in line with the in vivo results and they together confirmed that CEA‐CAR‐T cell in combination with rhIL‐12 had even better anti‐tumor effect than CEA‐CAR‐T cell treatment.

### 3.7 In vivo CEA‐CAR‐T cell persistence and cytokine release

**Figure 5:**
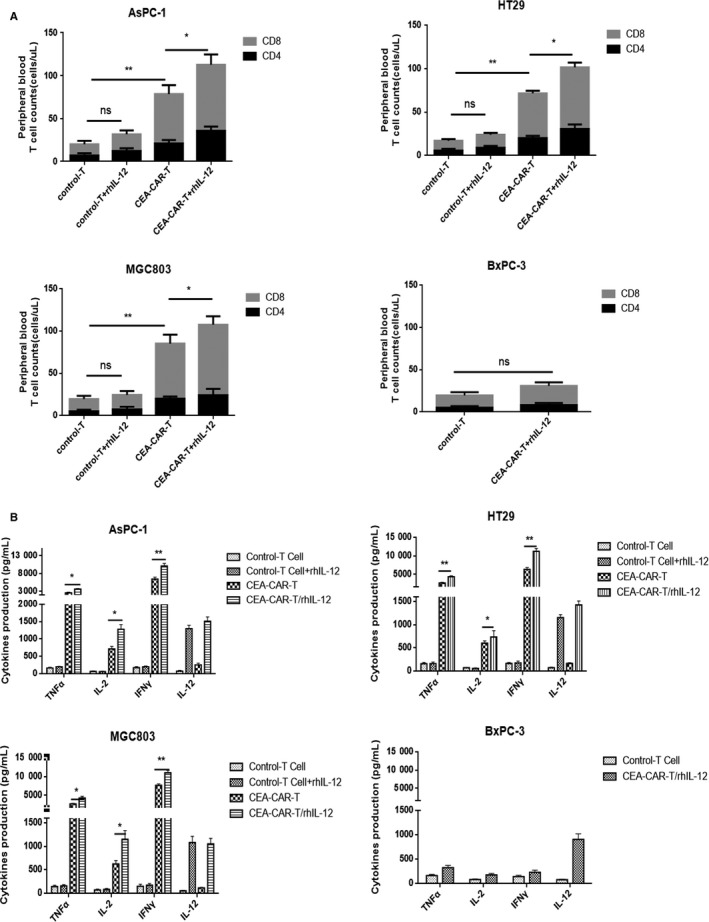
In vivo persistence and cytokine release levels of CEA‐CAR‐T cell in combination with rhIL‐12. (A) 14 d after effector cell infusion, 100 µL blood was collected and T‐cell number and CD8/CD4 T‐cell ratio were detected with flow cytometry analysis (n = 4, error bars denote standard deviation,*P < 0.05). (B) At the end of the experiment, blood levels of IL‐2, IL‐12, TNF‐α, and IFN‐γ were measured (n = 4, error bars denote standard deviation,*P < 0.05, **P < 0.01). CAR‐T, chimeric antigen receptor T; CEA, carcinoembryonic antigen

Previous research showed that the persistence of CAR‐T cells is in line with their anti‐tumor effect and high CD8 T cell to CD4 T cell ratio results in better therapeutic effect of adoptive cell therapy.^23^, ^24^ In the same animal work as shown in Figure 4A, we measured T‐cell number in mouse circulation and the ratio of CD8 T cell to CD4 T cell. As shown in Figure 5A, in mice injected with CEA‐positive tumor cells, the number of T cells and the ratio of CD8 T cell to CD4 T cell in CEA‐CAR‐T cell in combination with rhIL‐12 treatment group was higher than CEA‐CAR‐T cell treatment group. In addition, we detected the ratio of CEA‐CAR‐T cells in the samples of CEA‐CAR‐T treatment group and CAR‐CAR‐T cell in combination with rhIL‐12 treatment group. As shown in Figure S5, almost all T cells are CEA‐CAR‐T cells. From the above results, the higher number of T cells and CD8/CD4 ratio under CEA‐CAR‐T cell in combination with rhIL‐12 treatment was in line with their higher anti‐tumor activity.

Normally, the activation of CAR‐T cells in vivo will result in their killing of tumor cells accompanied with release of cytokines such as IL‐2, IFN‐γ, and TNF‐α. As shown in Figure 5B, in mice injected with CEA‐positive HT‐29‐RFP, AsPC‐1‐RFP, or MGC803‐RFP tumor cells and thereafter injected with CEA‐CAR‐T cells, the levels of serum cytokines were significantly increased compared with control‐T cell or control‐T cell in combination with rhIL‐12 treatment mice. Compared with CEA‐CAR‐T cell treatment mice, CEA‐CAR‐T cell in combination with rhIL‐12 resulted in a significant increase of serum IL‐2, IFN‐γ, and TNF‐α levels. And blood IL‐12 level was higher in mice of exogenous IL‐12 treatment groups. These changes of serum cytokine levels indirectly indicated the in vivo activity of CEA‐CAR‐T cells and were in line with the anti‐tumor activity of CEA‐CAR‐T cells under the corresponding treatment conditions.

The in vitro and in vivo experiments confirmed that rhIL‐12 can in vivo increase the anti‐tumor activity of CEA‐CAR‐T cells. Combination of CEA‐CAR‐T cells with rhIL‐12 may be an effective strategy in treatment of solid tumor.

## 4 DISCUSSION

Chimeric antigen receptor T cell therapy showed significant anti‐tumor activity in treatment of hematological cancers,^25^ however, expected anti‐tumor effect was limited in solid tumor treatment.^26^ Limited efficacy may result from the obstacles in tumor microenvironment.^27^ The inhibitory factors in TME inactivate the infiltrating CAR‐T cells and inhibit their anti‐tumor activity. Studies found that some cytokines can increase T‐cell activity, of which IL‐12 can mediate multiple immune reactions and some preclinical studies found that IL‐12 had anti‐tumor activity by regulation of immune reactions.^28^ Clinical studies found that intravenous injections of low dose rhIL‐12 resulted in significant anti‐tumor activity and the corresponding toxic effect was tolerable.^29^ Therefore, we used CEA‐CAR‐T cells in combination with rhIL‐12 to enhance their activity in treatment of solid tumors.

Carcinoembryonic antigen is a tumor‐associated antigen and is a promising target for CAR‐T cells to treat solid tumors.^30^ At the moment, there are four clinical trials with use of CEA specific CAR‐T cells (NCT03682744, NCT03818165, NCT02850536, NCT02349724). Therefore we used several cancer cell lines with high CEA expression, which include colorectal cancer HT‐29, pancreatic cancer AsPC‐1, and gastric cancer MGC803 cells, and tested the strong anti‐tumor activity of CEA‐CAR‐T cells in combination with rhIL‐12 against these cancer cell lines. The results confirmed that rhIL‐12 effectively activated CEA‐CAR‐T cells and increased the cytotoxic activity of CEA‐CAR‐T cells to CEA‐positive cancer cells. Furthermore, xenograft tumor model in nude mice was established with the individual human cancer cell lines that express fluorescence reporter protein and it was found that rhIL‐12 can in vivo increase the anti‐tumor activity of CEA‐CAR‐T cells and promote the proliferation of CEA‐CAR‐T cells in mice. A CEA‐negative cancer cell BxPC‐3 was also used in the in vitro and in vivo work and it was found that in this case the CEA‐specific CAR‐T cells cannot be activated and effectively kill the cancer cells. These works also showed that CEA is a promising target for CAR‐T cell treatment of cancer cells, especially treatment of solid tumors.

The results that rhIL‐12 can increase the anti‐tumor activity of CEA‐CAR‐T cells provide us a referential strategy to use other cytokines to strengthen the anti‐tumor activity of CAR‐T cells. Some potential cytokines can be used in this case. For instance, IL‐7 can regulate the homeostasis of naive T cells and memory T cells.^31^, ^32^, ^33^ It was also reported that IL‐7 can increase the number of circulating naive and memory T cells in SIV‐infected primates.^34^ Memory CAR‐T cell generation is reported to be crucial for the efficacy in the course of CAR‐T cell therapy.^35^ IL‐15 is another promising cytokine. It induces the proliferation of CD8 + T cells rather than Treg cells and effectively stimulates NK cells and T cells to exert their anti‐tumor activity.^36^ IL‐21 is also a promising cytokine with clinical application potential. In several tumor models, it induced the proliferation of CD8 + T cells and B cells after CD40 engagement.^37^ A phase II clinical trial in which IL‐21 is used to treat melanoma, renal carcinoma and non‐Hodgkin lymphoma is ongoing.^38^ Based on their in vivo functions, it is a promising strategy to use these cytokines in combination with CAR‐T cells.

Although we have used several in vitro and in vivo experiments to confirm that rhIL‐12 can increase the anti‐tumor activity of CEA‐CAR‐T cells, flaws exist. We have not differentially measured the changes of T‐cell subtype and have not investigated the mechanism for rhIL‐12 to strengthen CEA‐CAR‐T cells functions. The animal models we used are xerograph tumor model in nude mice and we did not use primary tumor model. These are the works we should do in the future. In general, we used several human tumor cell lines to establish animal models and confirmed that rhIL‐12 can increase the anti‐tumor activity of CEA‐CAR‐T cells and the combination use showed significantly better anti‐tumor activity than conventional CEA‐CAR‐T cells. These results strengthen our confidence to test whether other cytokines can increase CAR‐T cell activity, especially in treatment of solid tumors.

## CONFLICT OF INTEREST

The authors declare that they have no conflict of interest.

## Data Availability

The datasets generated in this study are available from the corresponding author on reasonable request.
