# Supplementary figures and images for: Significantly increased anti‐tumor activity of carcinoembryonic antigen‐specific chimeric antigen receptor T cells in combination with recombinant human IL‐12
Source: Cancer Med. 2019 Jun 25;8(10):4753–65. doi: 10.1002/cam4.2361 (PMC6712469; doi:10.1002/cam4.2361)

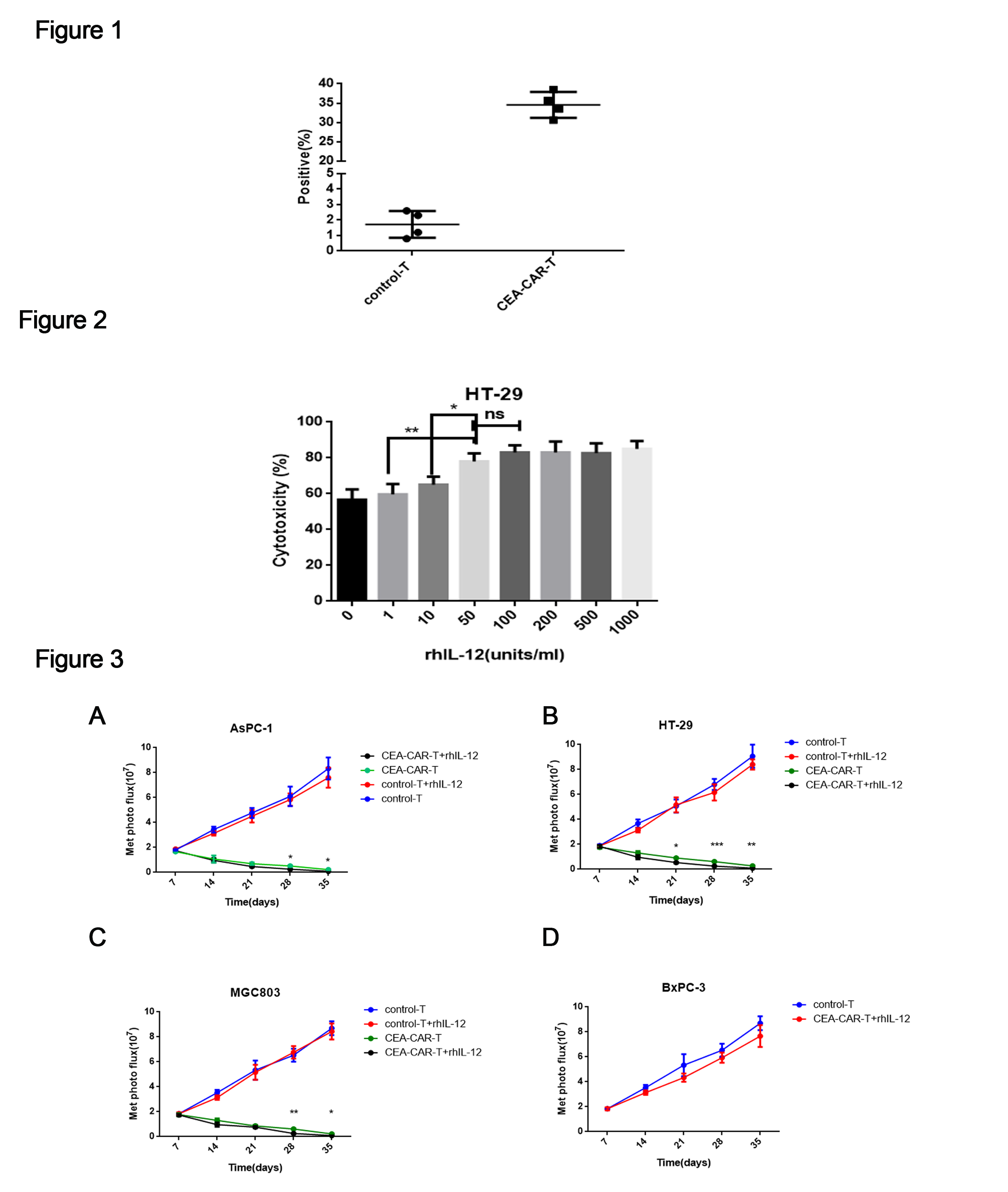

Supplement: Supplementary file 1 [file CAM4-8-4753-s001.tif]

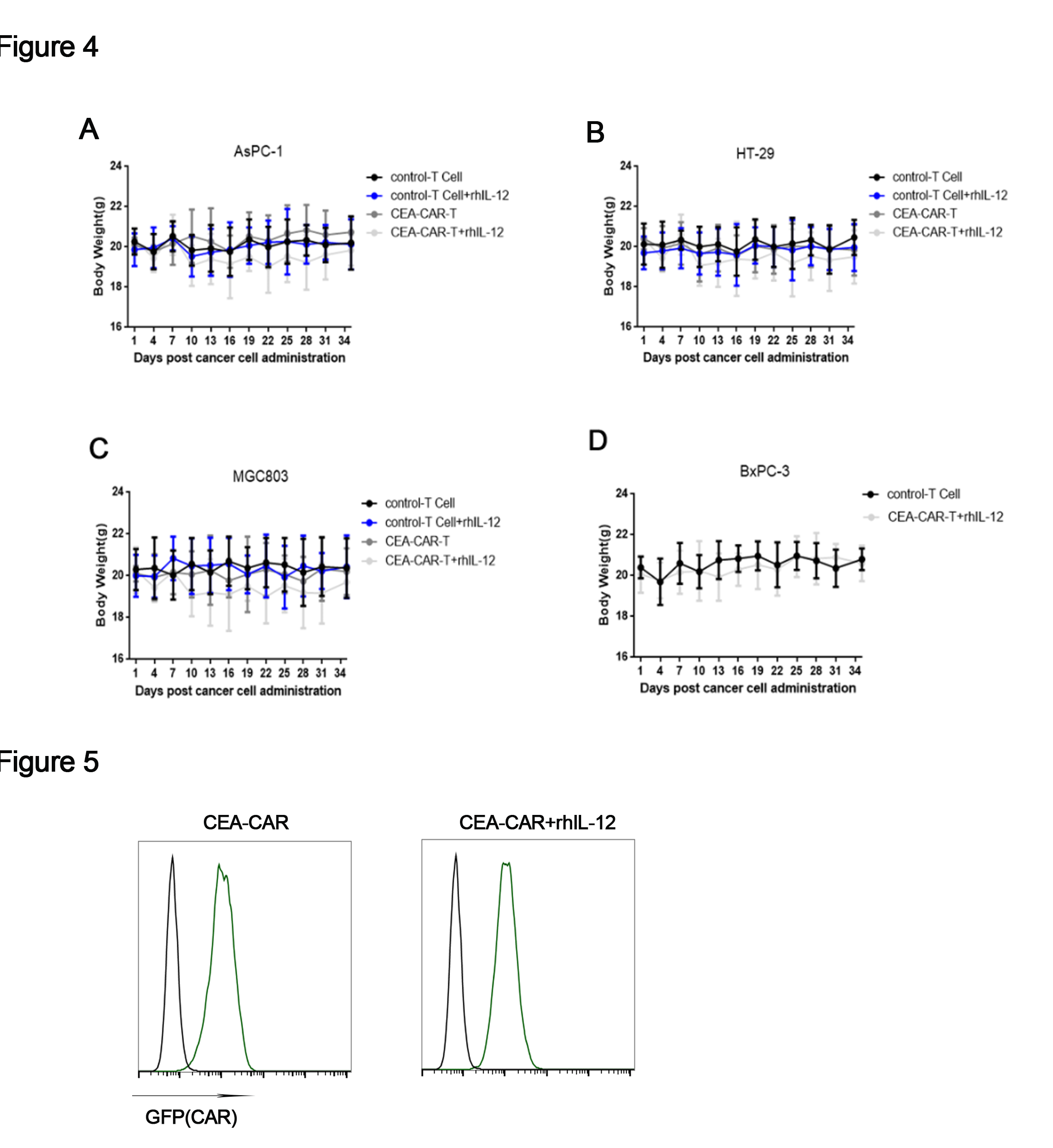

Supplement: Supplementary file 2 [file CAM4-8-4753-s002.tif]
